# Case report: Surgical and clinical results in bilateral lenticonus due to Alport syndrome

**DOI:** 10.22336/rjo.2025.64

**Published:** 2025

**Authors:** Esen Çakmak-Cengiz, Fadime Karaman-Atasever, Ertuğrul Can

**Affiliations:** 1 Ünye State Hospital, Ordu, Turkey; 2 Gaziosmanpaşa Education and Research Hospital, Istanbul, Turkey; 3 Department of Ophthalmology, Ondokuz Mayis University Hospital, Samsun, Turkey

**Keywords:** Alport syndrome, anterior lenticonus, phacoemulsification, AS = Alport syndrome, PHACO = Phacoemulsification, D = Diopters, IOL = Intraocular lens, BCVA = Best-corrected visual acuity, OCT = Optical coherence tomography, VEM = Viscoelastic material, CCC = Continuous curvilinear capsulorhexis

## Abstract

Alport syndrome (AS) is a genetic disease characterized by hereditary nephritis, sensorineural hearing loss, and ocular anomalies. The most common ocular findings associated with AS include anterior lenticonus, retinal dot-fleck configuration, and corneal posterior polymorphism. Anterior lenticonus is 8 times more common in men with AS than in women. This case report evaluated the clinical and optical results obtained after phacoemulsification (PHACO) surgery and intraocular lens (IOL) implantation performed for bilateral anterior lenticonus due to AS. A 35-year-old male patient had complained of progressively decreasing vision for 10 years. The patient’s ophthalmologic examination revealed bilateral anterior lenticonus and nuclear sclerosis. Continuous curvilinear capsulorhexis was applied in the anterior capsulorhexis method. Phacoemulsification surgery was performed using a high-viscoelastic material, starting the anterior capsulorhexis from the mid-periphery. The surgery was completed without anterior and posterior capsular rupture. Optimal postoperative refraction was achieved due to the IOL calculation performed using the traditional SRK-T formula. As a result, it has been shown that successful and uncomplicated results can be achieved with careful surgical technique and appropriate IOL selection in anterior lenticonus surgery due to AS.

## Introduction

The clinical triad of Alport syndrome (AS) is hereditary nephritis, sensorineural deafness, and ocular abnormalities [1]. The primary defect in Alport syndrome is in the synthesis of type 4 collagen, and anomalies occur in organs where type 4 collagen is found in the basement membrane structure [2]. Ocular findings are more common in patients carrying an X-linked mutation. Corneal posterior polymorphism, anterior lenticonus, and retinal dot-fleck configuration are frequently associated. Anterior lenticonus is 8 times more common in men with AS than in women [1]. Researchers from various groups have recently reported that temporal retinal thinning is a frequently observed ophthalmological feature in AS [3]. Anterior lenticonus can easily be overlooked when the pupil is not dilated. This case report presents our experience with clinical and optical results of uncomplicated phacoemulsification (PHACO) surgery for bilateral lenticonus.

## Case report

**Fig. 1:**
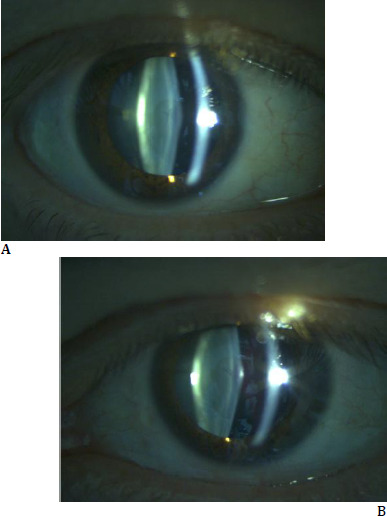
**A, B** On biomicroscopy examination with dilated pupil (Haag Streit Eye Suite imaging system connected to BQ900 slit-lamp biomicroscope), anterior lenticonus and nuclear cataract are observed in both eyes

**Fig. 2:**
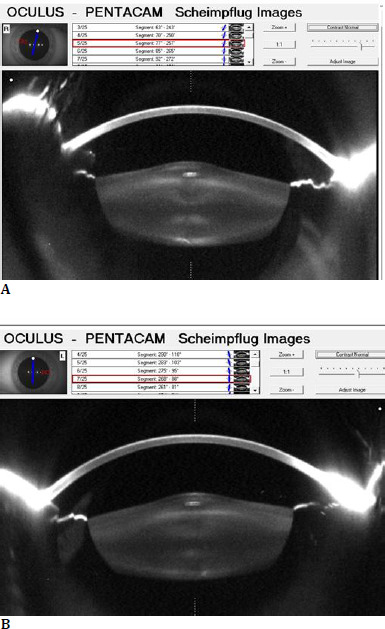
**A, B** Pentacam Scheimpflug camera (OCULUS, Wetzlar, Germany) image supporting the clinical findings of anterior lenticonus

**Fig. 3:**
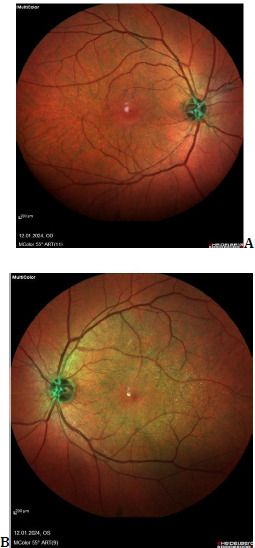
**A, B** Although present in both eyes, the dot-fleck appearance in the temporal macula of the left eye is shown in fundus multicolor imaging (Spectralis OCT, Heidelberg Instruments)

**Fig. 4:**
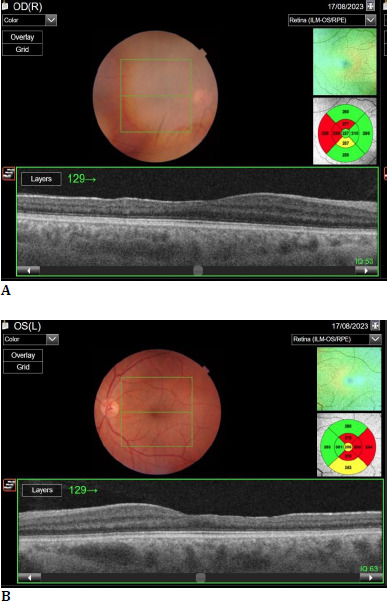
**A, B** Retinal thinning is seen in the temporal macula of both eyes on optical coherence tomography (Topcon DRI-OCT Triton Swept-Source OCT)

A 35-year-old male patient presented to an ophthalmology clinic with complaints of decreased vision in both eyes that had been present for approximately 10 years and had progressed over time. The patient was diagnosed with AS when he was six years old. During the patient’s systemic examination, it was found that he developed renal failure in his teens and then hearing loss. He underwent a kidney transplant due to end-stage renal failure at the age of seventeen. In his ophthalmological examination, with autorefractometry after cycloplegia, right eye refraction was -14.50-3.75 x 170, left eye - 10.50-6.50 x 180. Steep and flat keratometry values: right eye steep keratometry was 47.2 x 95 diopters (D) and flat keratometry was 46.7 x 5 D, and left eye steep keratometry was 48.3 x 83 D and flat keratometry was 47.3 x 173 D. It was observed that lenticular astigmatism contributed more to manifest refraction than corneal astigmatism. Axial length was 22.12 mm in the right eye and 22.02 mm in the left eye. The best-corrected visual acuity (BCVA) was 20/50 in the right eye and 20/40 in the left eye. Intraocular pressures were 12 mmHg in the right eye and 15 mmHg in the left eye. After dilation with cycloplegic, anterior lenticonus and nuclear sclerosis were observed in both eyes on biomicroscopic examination (**Fig. 1A, B**). The anterior coning of the lens and anterior chamber narrowing in both eyes were recorded with the Pentacam Scheimpflug Camera (OCULUS, Wetzlar, Germany) (**Fig. 2A, B**). Fundus examination revealed a retinal dot fleck configuration in the temporal macula in both eyes, prominent in the left eye (**Fig. 3A, B**). There was temporal retinal thinning in optical coherence tomography (OCT) (Topcon DRI-OCT Triton Swept-Source OCT) in both eyes (**Fig. 4A, B**).

The aim was to achieve emmetropic refraction after surgery, and IOL calculation was performed using the SRK-T method. The informed consent form for the surgical procedure was obtained from the patient. Cataract surgery was performed on both eyes one week apart, starting from the poorly seeing eye under general anesthesia. Viscoelastic material (VEM) containing cohesive sodium hyaluronate was used. Anterior capsulorhexis was performed using the continuous curvilinear capsulorhexis (CCC) method. No cortex evacuation was performed before anterior capsulorhexis was carefully performed with manual utrata forceps without staining the anterior capsule and starting from the midperiphery. Lens extraction was performed with the PHACO method, and an aspheric IOL (Sensar AR40, Johnson & Johnson) with an optical power of +21.5 D in the right eye and +20.0 D in the left eye was implanted into the capsular bag. VEM was removed with bimanual irrigation and aspiration. No anterior or posterior capsule rupture was observed. Autorefraction in the postoperative 1st month examination was +0.50 +0.75 axis 97 in the right eye and +0.50 -1.0 axis 15 in the left eye. The patient’s vision was full in both eyes without correction.

## Discussion

Alport syndrome is found in 1/5000 of the population [4]. In men, kidney failure and high-level sensorineural hearing loss usually occur in their twenties. Ocular anomalies are associated with Alport syndrome in 11% to 90% of cases [4]. The most common ocular findings are retinal dot-fleck retinopathy (85%), anterior lenticonus (25%), and rarely corneal posterior polymorphism [5,6]. Anterior lenticonus usually occurs after renal failure. The presence of dot-fleck retinopathy in any individual and a family history of AS or renal failure provide a diagnosis of AS [7]. If the retinal lesion does not involve the macula, it is not responsible for decreased vision. In this case, the temporal macula had a bilateral retinal dot-fleck configuration and retinal thinning. In this case, surgical technique, IOL calculation, and postoperative refractive outcome were evaluated.

After CCC and PHACO, surgery was performed on both eyes of the patient, starting from the midperiphery instead of the anterior pole, and from the eye with the worse vision. A 3-piece foldable IOL was implanted in the capsular bag, starting from the worse visual eye, and an excellent long-term visual prognosis was achieved. The central pole of the anterior capsule is more fragile than the midperiphery, and spontaneous tears may occur in the more fragile anterior capsule during anterior capsulorhexis in patients with AS [6,8]. In both eyes, anterior capsulorhexis was started from the midperiphery, and manual utrata capsule forceps were used. With high care and surgical experience, we can avoid complications such as capsule extension to the periphery and notching [1]. Since high intralenticular pressure was not considered, the cortical material was not evacuated before capsulorhexis. However, publications report that evacuating cortical material before anterior capsulorhexis reduces the risk of complications [9]. There were no problems during the PHACO and irrigation-aspiration stages. Since the patient was young, a 3-piece acrylic hydrophobic foldable IOL implanted in the posterior chamber was preferred due to good stabilization and a lower probability of posterior capsule opacification. There was an anterior chamber narrowing due to anterior lenticonus. IOL calculation with the traditional SRK-T formula resulted in optimal postoperative refraction. In anterior lenticonus, successful surgical and visual results can be obtained with the femtosecond laser-assisted PHACO method.

In contrast, successful visual and surgical results can be obtained with the classical PHACO method [10,11]. In these cases, we recommend performing an anterior capsulorhexis starting from the midperiphery, followed by standard phacoemulsification and posterior chamber IOL implantation. When intralenticular pressure increase is not considered, successful surgery can be performed without preferring anterior capsule staining and cortex material evacuation before anterior capsulorhexis.

## Conclusion

In this case, PHACO surgery and intraocular lens implantation were performed for bilateral anterior lenticonus due to Alport syndrome, resulting in a successful visual outcome. Starting the anterior capsulorhexis from the midperiphery during surgery effectively prevented capsular ruptures. Intraocular lens calculation using the traditional SRK-T formula provided optimal postoperative refractive results. It has been demonstrated that uncomplicated and successful outcomes can be achieved through careful surgical technique and the selection of appropriate lenses in anterior lenticonus surgery.
